# Cigarette Smoking during Pregnancy: Effects on Antioxidant Enzymes, Metallothionein and Trace Elements in Mother-Newborn Pairs

**DOI:** 10.3390/biom10060892

**Published:** 2020-06-10

**Authors:** Alica Pizent, Maja Lazarus, Jelena Kovačić, Blanka Tariba Lovaković, Irena Brčić Karačonji, Tanja Živković Semren, Ankica Sekovanić, Tatjana Orct, Karmen Branović-Čakanić, Nataša Brajenović, Andreja Jurič, Iva Miškulin, Lana Škrgatić, Sandra Stasenko, Tatjana Mioč, Jasna Jurasović, Martina Piasek

**Affiliations:** 1 Institute for Medical Research and Occupational Health, 10000 Zagreb, Croatia; apizent@imi.hr (A.P.); jkovacic@imi.hr (J.K.); btariba@imi.hr (B.T.L.); ibrcic@imi.hr (I.B.K.); tzivkovic@imi.hr (T.Ž.S.); asekovanic@imi.hr (A.S.); torct@imi.hr (T.O.); nbrajen@imi.hr (N.B.); ajuric@imi.hr (A.J.); jurasovic@imi.hr (J.J.); mpiasek@imi.hr (M.P.); 2 Croatian Veterinary Institute, 10000 Zagreb, Croatia; branovic@veinst.hr; 3 University Hospital Centre, 10000 Zagreb, Croatia; iva.miskulin@gmail.com (I.M.); lana.skrgatic@mef.hr (L.Š.); 4 Merkur University Hospital, 10000 Zagreb, Croatia; sandra.stasenko@zg.t-com.hr (S.S.); tatjana.mioc@hotmail.com (T.M.)

**Keywords:** toxic metals, essential elements, SOD, GPx, metallothionein, sparse discriminant analysis, developmental exposure, maternal smoking

## Abstract

The effect of maternal smoking as a source of exposure to toxic metals Cd and Pb on superoxide dismutase (SOD) and glutathione peroxidase (GPx) activity, metallothionein (MT), Cd, Pb, Cu, Fe, Mn, Se and Zn concentrations were assessed in maternal and umbilical cord blood and placenta in 74 healthy mother-newborn pairs after term delivery. Sparse discriminant analysis (SDA) was used to identify elements with the strongest impact on the SOD, GPx and MT in the measured compartments, which was then quantified by multiple regression analysis. SOD activity was lower in maternal and cord plasma, and higher in the placenta of smokers compared to non-smokers, whereas GPx activity and MT concentration did not differ between the groups. Although active smoking during pregnancy contributed to higher maternal Cd and Pb concentrations, its contribution to the variability of SOD, GPx or MT after control for other elements identified by SDA was not significant. However, an impaired balance in the antioxidant defence observed in the conditions of relatively low-to-moderate exposure levels to Cd and Pb could contribute to an increased susceptibility of offspring to oxidative stress and risk of disease development later in life. Further study on a larger number of subjects will help to better understand complex interactions between exposure to toxic elements and oxidative stress related to maternal cigarette smoking.

## 1. Introduction

There is emerging evidence that maternal exposure to various environmental stressors may have an important role in programming the susceptibility of offspring to adverse health effects. Foetal programming includes permanent structural, physiological and metabolic adaptations to the intrauterine environment, which is the first environment of a developing foetus. Any changes during the perinatal period can increase the risk of developing a disease later in life. This concept is referred to as the developmental origins of health and disease (DOHaD) [1,2]. The mechanisms of the health consequences due to environmental exposures and conditions manifested postnatally may be oxidative stress, which is recognised as one of the key mediators included in the programming of future health and diseases of the offspring [3,4].

Cigarette smoking during pregnancy is one of the leading environmental factors that can adversely affect the health of women during the reproductive period and her progeny. Despite efforts to decrease cigarette smoking, globally, 52.9% (95% CI 45.6–60.3) of women who smoked daily continued to smoke during and/or after the pregnancy [5]. A smoking pregnant woman exposes her embryo/foetus to a variety of chemicals from tobacco smoke as well as to increased risk of detrimental effects, directly via placental transfer of the toxicants and indirectly by affecting placental vasculature, umbilical artery blood flow, and impaired placental nutrient passage [6,7]. 

Tobacco smoke contains a vast number of chemicals related to the initiation or development of pathologic processes affecting foetoplacental development and pregnancy outcomes [7]. Among them are free radicals and reactive oxygen species (ROS) with the potential to cause oxidative damage to cellular membrane lipids, proteins, enzymes and DNA [8]. Excessive ROS may promote the development of an abnormal placental vascular network and functions [9,10], contributing to impaired nutrient and oxygen transport to the developing foetus [11,12]. In smoking mothers and their newborns, increased lipid peroxidation and impaired prooxidant-antioxidant balance were reported [12,13,14]. In addition, smoking is, except food, the main source of cadmium (Cd) and lead (Pb) in the general population in unpolluted areas [15,16]. Both toxic metals were shown to adversely affect human health, especially during periods of particular sensitivity, such as pregnancy and lactation (perinatal period) [17]. Blood levels reflect recent Cd and Pb exposure, while the placenta mirrors both past and recent exposures due to element redistribution during pregnancy [7]. Although redox inactive elements, Cd and Pb can contribute to oxidative stress by replacing essential elements from binding site(s) in various cytoplasmic and membrane proteins, affecting their structure, activity and function [18,19]. 

Under normal conditions, the antioxidant system minimizes the overproduction of reactive species and prevents the adverse effects of oxidative stress. Antioxidant enzymes superoxide dismutase (SOD) and glutathione peroxidase (GPx) are at the first line of defence against ROS and its by-products. For their optimal activity and structure, trace quantities of essential elements are required: copper (Cu) and zinc (Zn) for Cu,Zn-SOD (in the cytoplasm and extracellularly), manganese (Mn) for Mn-SOD (in the mitochondria) and selenium (Se) for Se-GPx (in the cytoplasm and the mitochondria). These elements are also essential for embryogenesis and foetal growth and development [20]. 

Metallothioneins (MT), small cysteine-rich metal binding proteins, have an important role in Zn and Cu storage and homeostasis. Its additional important role is scavenging free radicals generated in oxidative stress and detoxification. Due to many -SH groups and high affinity/high capacity to bind various reactive metal ions, such as Cd, mercury (Hg), Cu, Pb, nickel (Ni), cobalt (Co), iron (Fe), silver (Ag) and gold (Au), MT contribute to their decreased availability for toxic effects in conditions of acute exposure, whereas in conditions of chronic exposure accumulated toxic metals (such as Cd-MT or Hg-MT complex) may pose a risk for toxic effects (reviewed in [21,22]). Tobacco smoking can affect MT expression in the placenta and subsequent binding, transfer and placental accumulation of related metal ions. However, the relevant literature lacks studies combining smoking-related effects on MT in healthy mother-newborn pairs [23] and antioxidant capacity taking into account the interplay of multiple elements [19,24]. As both antioxidant enzymes and MT share important roles in element physiology and smoking-related toxicology, as well as in the maintenance of pro/antioxidant balance, it is of the outmost significance to follow those parameters simultaneously. 

The aim of this study was to assess the effect of cigarette smoking and related exposure to toxic elements on antioxidant enzymes, MT and essential element concentrations in samples of maternal and foetal origin taken immediately after spontaneous vaginal delivery at term in healthy mother-newborn pairs from Croatia. We measured the activity of SOD and GPx in maternal and umbilical cord plasma, concentration of MT, Cu, Fe, Se and Zn in maternal and cord serum and Cd, Mn and Pb in maternal and cord blood. All these parameters were also determined in the placenta. We applied an innovative approach of identifying elements with the strongest impact on SOD, GPx and MT variability by using sparse discriminant analysis followed by multiple regression analyses.

## 2. Materials and Methods

### 2.1. Study Participants

The study was carried out in 74 mother-newborn pairs selected within a wider study on a larger cohort recruited in the period between December 2017 and January 2019. Participants were healthy postpartum women who gave normal vaginal birth at term (37th–42th week of pregnancy) in the maternity wards of two clinical hospitals in Zagreb, Croatia. Each participant was informed about the study aim and protocol and signed an informed consent to willingly participate in the study. The study was approved by the ethics committees of three collaborating institutions: University Hospital Centre (no. 021-1/43-18), Merkur University Hospital (no. 03/1-5102/1) and the Institute for Medical Research and Occupational Health (no. 100-21/17-07) in Zagreb, Croatia. Relevant data regarding medical history, sociodemographic characteristics, self-declared cigarette smoking, as well as maternal and neonatal clinical data were collected by a questionnaire as described in our previous studies [7,25] and from clinical records. Based on self-reported information about cigarette smoking habits, the two studied groups were planned to be designated based upon the following criteria: non-smokers—never smoked or smoked >12 months before the last pregnancy, and smokers—smoking any time during pregnancy or within 12 months before last pregnancy. To ensure an objective assessment of smoking habit in the study participants, smoking status was additionally quantified by urinary cotinine measurement. Several studies have suggested that cut-off urinary cotinine level of 25–50 ng/mL is appropriate to identify smokers among pregnant and postpartum women [26,27]. In order to distinguish active smokers from non-smokers, the cut-off point was set up at 100 ng/mL. A urinary cotinine level lower than the limit of quantification (LOQ) was set as the cutpoint for non-smokers to avoid misclassification between non-smokers and passive smokers who were involuntarily exposed to the low-level environmental tobacco smoke. Accordingly, two study groups were identified and compared: non-smokers (*n* = 37) with urinary cotinine < LOQ, and smokers (*n* = 37) with urinary cotinine ≥ 100 ng/mL.

### 2.2. Sample Collection

Spot urine samples from pregnant women were collected in a screw-cap container on the day of hospitalization for delivery. Non-fasting maternal and umbilical cord blood samples were taken within 10 min after delivery. Blood was collected in BD vacutainer for trace element testing tubes with K_2_EDTA for whole blood and plasma analyses and without anticoagulant for serum analyses. Whole placenta with umbilical cord was placed in zip-lock polyethylene bags after delivery. All samples were stored at 4 °C until transport to the analytical laboratory. Blood and placental samples were further processed within two hours after delivery. 

In the analytical laboratory, urine samples were decanted avoiding visible particles, aliquoted into screw-cap vials and stored until analysis at −20 °C. Whole blood, plasma and serum samples were aliquoted and stored at −80 °C until analysis. Fresh placentas were sampled following the method described earlier [7,25]. Whole placentas were placed on the maternal side, blotted on the filter paper, umbilical cord and extraembryonic membranes were trimmed off and the fresh placental mass recorded. Using a ceramic knife, three full-thickness sections (including maternal and foetal surfaces) were taken from each placenta. One central (C) section was taken from the midline of the placental disc avoiding the region of umbilical cord insertion. Two peripheral (P) sections were taken between the central region and periphery, outermost 3 cm of the outer placental disk margin. The decidua basalis and the chorionic plate of the placenta were then cut off from each sample, leaving the trophoblastic tissue (chorionic villi) for further preparation. Representative samples were further collected by cutting C and P sections into small cubes of approximately 1–2 g fresh tissue weight and subsamples consisting of one C or two P sections from each placenta were stored in cryotubes at −80 °C until analysis. 

### 2.3. Superoxide Dismutase Analyses

SOD activity was measured in plasma and supernatant of placental tissue homogenates using Superoxide Dismutase Assay Kit (item no. 706002, Cayman Chemicals, Ann Arbor, MI, USA). Placental C and P subsamples were homogenized respectively in 5 mL of cold 20 mM HEPES buffer, pH 7.2, containing 1 mM ethylene glycol tetraacetic acid (EGTA), 210 mM mannitol and 70 mM sucrose per gram tissue with Precellys^®^ Evolution (Bertin Technologies, Montigny-le-Bretonneux, France) tissue homogenizer. Homogenates were then centrifuged (Eppendorf Centrifuge 5417 R, Eppendorf AG, Hamburg, Germany) at 10,000× *g* for 15 min at 4 °C and supernatant was stored at −80 °C until analysis. The absorbance was read at 445 nm using a plate reader Tecan Infinite M200 Pro (Tecan Group Ltd., Männedorf, Switzerland). One unit of SOD is defined as the amount of enzyme needed to exhibit 50% dismutation of the superoxide radical. Activity of SOD was expressed as U/mL of plasma and U/g of proteins in the supernatant of placental tissue homogenates, respectively.

### 2.4. Glutathione Peroxidase Analyses

GPx activity was measured in plasma and supernatant of placental tissue homogenates using Glutathione Peroxidase Assay Kit (item no. 703102, Cayman Chemicals, Ann Arbor, MI, USA). Placental C and P subsamples were homogenized respectively in 5 mL of cold buffer containing 50 mM Tris-HCl, pH 7.5, 5 mM ethylenediaminetetraacetic acid (EDTA), and 1 mM dithiothreitol (DTT) per gram tissue with the OMNI TH Tissue Homogenizer (OMNI International, Inc., Kennesaw, GA, USA). Homogenates were then centrifuged (Eppendorf Centrifuge 5417 R, Eppendorf AG, Hamburg, Germany) at 10,000× *g* for 15 min at 4 °C and supernatant was stored at −80 °C until analysis. The absorbance was read at 340 nm using a plate reader Tecan Infinite M200 Pro (Tecan Group Ltd., Männedorf, Switzerland). One unit is defined as the amount of enzyme that will cause the oxidation of 1.0 nmol of NADPH to NADP^+^ per minute at 25 °C. Activity of GPx was expressed as nmol/min/mL of plasma and nmol/min/g of proteins in supernatant of placental tissue homogenates, respectively.

### 2.5. Metallothionein Analyses

Metallothionein-2 isoform was measured in serum and placental supernatant. We decided to follow the level of MT2 isoform of the protein as the main MT isoform among four [28] and as Ronco et al. [29] found placental MT2, and not the MT1 isoform, to increase in smokers compared to non-smokers. First, central (C part) and two peripheral (together as P part) placental parts were thawed and respectively homogenized (10% *w*/*v*) in 0.01 mol/L PBS buffer with Precellys^®^ Evolution (Bertin Technologies, Montigny-le-Bretonneux, France) tissue homogenizer. Homogenates were then centrifuged (Eppendorf Centrifuge 5417 R, Eppendorf AG, Hamburg, Germany) at 5000× *g* at 4 °C for 15 min and the supernatant was stored at −80 °C until MT2 analysis. The concentration of MT2 was quantified at 450 nm on a microplate reader (Infinite F50, Tecan Trading AG, Männedorf, Switzerland) using a commercial kit (enzyme-linked immunosorbent assay (ELISA) kit, SEB868Hu 96 tests, Cloud-Clone Corp., Houston, TX, USA). The numeric MT2 data were expressed in ng/g protein or in ng/mL serum.

### 2.6. Protein Analysis

Total protein in the supernatant of placental homogenates was determined using the standard Bradford colorimetric assay with bovine serum albumin as the standard [30].

### 2.7. Trace Element Analyses

Levels of Cd, Mn and Pb in maternal and cord whole blood and Cu, Fe, Se and Zn in maternal and cord serum were measured after dilution of samples with alkaline solution (0.7 mmol/L ammonia, 0.01 mmol/L EDTA, 0.07% (*v*/*v*) Triton X-100, and 3 μg/L of internal standard. Partially thawed placental C and P part (0.5 g) were acid digested in an UltraCLAVE IV (Milestone, Sorisole, Italy) microwave digestion system before the elements were quantified by means of inductively coupled plasma mass spectrometry (ICP-MS; Agilent 7500cx, Tokyo, Japan) following the procedure detailed elsewhere [31]. Element concentrations were measured in the C and P part of placenta to obtain homogeneous sample and average placental element concentrations were used in the following analyses. The accuracy of measurements was controlled using blood and tissue standard (SRM) and certified reference materials (CRM): Seronorm^TM^ Trace Element Blood (Levels 1, 2 and 3), Seronorm^TM^ Trace Elements Serum (Levels 1 and 2) (Sero AS, Billingstad, Norway), ClinChek^®^ Whole Blood Control (Levels 1, 2 and 3), and ClinChek^®^ Serum Control (Levels 1 and 2) (Recipe, München, Germany), BCR-185R Bovine Liver, BCR-186R Pig Kidney (IRMM, Geel, Belgium), and SRM 1577b Bovine Liver (NIST, Gaithersburg, MD, USA). The limit of detection (LOD) and the results of the element analyses in these reference materials performed for quality control of the measurements were presented in the Supplementary Tables S1 and S2. Overall recoveries were 92–113% of the assigned analytical values. The measured elements were expressed in µg/L serum or whole blood and mg/kg of wet placental tissue weight. 

### 2.8. Cotinine Analysis

Cotinine as a marker of exposure to tobacco smoke was extracted by 85 μm polyacrylate fiber (Supelco, Bellefonte, USA) exposed to headspace above 1 mL of maternal urine at 80 °C for 15 min (headspace-solid phase microextraction; HS-SPME). Quantification was obtained by gas chromatography-mass spectrometry (GC-MS; Varian 3400 CX gas chromatograph equipped with a Saturn 4D ion trap mass spectrometer) under conditions described previously by Brčić Karačonji et al. [32] and data expressed in ng/mL. The LOQ for urinary cotinine was 0.25 ng/mL.

### 2.9. Statistical Analyses

The Shapiro–Wilk test was used to assess normality of data. Accordingly, the parametric Student’s *t* test or nonparametric Mann–Whitney *U* test was performed to detect differences in parameters between non-smoking and smoking mothers and their newborns. Differences in SOD and GPx activity, MT and element concentrations between maternal and neonatal compartments were tested with the Wilcoxon matched-pairs test. Categorical variables were analysed by Fisher’s test. Results were presented as a mean ± standard deviation or median and range. The Spearman correlation coefficients were used to evaluate the statistical associations between variables of interest. Prior to further analyses, dependent variables were transformed to approach normal distribution by adding a constant (only for SOD in placenta and MT in maternal serum) and taking the natural logarithm. 

First, associations of Cd and Pb with SOD, GPx, and MT were assessed in linear regression models adjusted for age, education, and smoking status (omitted in the case of Cd to avoid problems with collinearity). 

In the second part of the study, we assessed the simultaneous effects of selected elements on SOD, GPx, and MT in measured matrices. To identify elements with the strongest associations with SOD, GPx, and MT, sparse discriminant analysis (SDA), especially suitable for small samples with a large number of variables [33] was used. Unlike simpler analyses, such as correlation or multiple regression analysis that can assess the contribution of a limited number of predictors at the same time due to sample size constraints, SDA analysis considers the effect of all potential predictors simultaneously. Thus, this analysis allows us to assess whether Cd or Pb as markers of smoking exposure are among the elements contributing most significantly to differences in MT and antioxidant enzymes when considering simultaneous exposure to selected elements. After log-transformation, all data columns used as predictors (elements) were normalised to have a zero mean and unit length. Dependent variables (SOD, GPx, MT) were categorised into three groups according to the tertiles of observed data. As potential predictors, Cd, Mn and Pb in maternal and cord blood, and Cu, Fe, Se and Zn in maternal and cord serum, generally accepted biomarkers of their exposure were considered. In addition, all seven elements in the placenta were included. Since the number of variables that can be included in the further multivariate analyses is limited by the sample size, number of potential predictors was set to four. Tuning parameter λ was selected by the leave-one-out cross-validation procedure [33].

As SDA output does not assess the model significance, further multiple regression analyses were performed following SDA analyses. Variables considered as predictors in these regression models were four elements from the SDA analysis, and mother’s age, smoking and education. When Cd was selected as the predictor, smoking was omitted to reduce the effect of collinearity. 

All analyses were performed in statistical software R, version 3.5.0 (R Foundation for Statistical Computing, Vienna, Austria).

## 3. Results

### 3.1. Maternal and Neonatal General Characteristics

Table 1 shows the general characteristics of the study participants. The average maternal age was 31, and smoking mothers were younger than non-smokers (30 vs. 33 years). According to the self-reported data on smoking habits, three participants declared as non-smokers had urinary cotinine level higher than the cut-off level of 100 ng/mL (i.e., 199 ng/mL, 556 ng/mL and 1160 ng/mL, respectively). Therefore, they were considered smokers. Participants self-declared as smokers reported to smoke 10 (2–20) cigarettes per day before pregnancy and 9 (1–20) cigarettes per day during pregnancy, and only three reported to smoke 20 cigarettes per day during the entire pregnancy. No significant differences between smokers and non-smokers were found in parity, body mass index (BMI) before pregnancy, BMI before delivery, and weight gain during pregnancy. About 70% of newborns were male. There was an equal sex distribution of newborns between smokers and non-smokers. All newborns were of excellent health with the highest median value of APGAR score 10, both at minutes 1 and 5 (data not shown).

### 3.2. Comparison of Measured Parameters Between Maternal and Neonatal Compartments

#### 3.2.1. Element Concentrations

**Figure 1:**
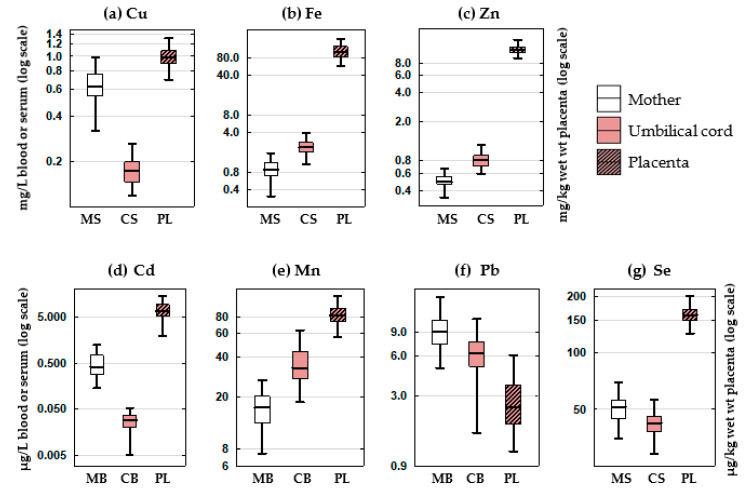
Concentration of (**a**) Cu, (**b**) Fe, (**c**) Zn, (**d**) Cd, (**e**) Mn, (**f**) Pb and (**g**) Se in the samples of maternal and umbilical cord blood and serum and placenta of 74 mother-newborn pairs. The boxes and lines represent 25%–75% interquartile range and median, respectively, and whiskers represent non-outlier range. Abbreviations: MB—maternal blood, MS—maternal serum, CB—cord blood, CS—cord serum, PL—placenta.

The concentrations of elements measured in maternal and cord blood or serum and in the placenta are presented in Figure 1 and Supplementary Table S3. 

When comparing the levels measured in the maternal with umbilical cord blood or serum, a significant difference was observed for most of the elements, regardless of maternal smoking habit. The concentrations in the cord blood were significantly lower than in maternal blood for Cd and Pb, and higher for Mn. The concentrations in the cord serum were significantly lower than in maternal serum for Cu and Se, and higher for Fe and Zn.

#### 3.2.2. SOD and GPx Activity and MT Concentration

When comparing the levels between maternal and umbilical cord blood plasma, a significant difference was found for both enzymes, regardless of maternal smoking habit. The median of SOD activity was 1.7-fold higher and of GPx activity 1.6-fold lower in cord than in maternal plasma of respective mothers. 

The MT in cord sera was 11 times higher than in the sera of respective mothers. No significant difference was found for SOD, GPx and MT between the central and peripheral compartment of placenta. Therefore, the average values of these parameters in central and peripheral placental compartments were used in further statistical analyses.

### 3.3. Comparison of Measured Parameters between Smokers and Non-Smokers

#### 3.3.1. Element Concentrations

The measured concentrations of elements were compared between smoking and non-smoking group (Table S3). Smoking mothers had significantly higher median Cd concentration in their blood (0.683 vs. 0.307 µg/L) and placenta (8.07 vs. 6.33 µg/kg wet w.t.) compared to non-smokers, whereas no significant difference in cord blood Cd (0.029 vs. 0.028 µg/L) was found. Although Pb was higher in smokers than in non-smokers in all of the measured compartments (maternal blood: 9.32 vs. 8.30 µg/L, cord blood: 6.55 vs. 6.07 µg/L, placenta: 2.86 vs. 2.07 µg/kg wet w.t., respectively), only the difference in placenta was statistically significant. 

Smoking mothers had significantly lower Fe in serum (0.760 vs. 1.058 mg/L) and placenta (94.4 vs. 108.8 mg/kg wet w.t.), Se in cord serum (40.4 vs. 43.6 µg/L), and higher Zn in placenta (11.1 vs. 10.7 mg/kg wet w.t.) than non-smoking mothers. No significant difference between smokers and non-smokers was found for Cu and Mn in all measured matrices, Fe in cord serum, Se in maternal serum and placenta, and Zn in maternal and cord serum.

#### 3.3.2. SOD and GPx Activity and MT Concentration

The levels of SOD, GPx and MT were compared between smoking and non-smoking group (Table 2). Activity of SOD was significantly lower in maternal and umbilical cord plasma and 1.7-fold higher in placenta of smoking than non-smoking group. Activity of GPx was similar in maternal and cord plasma and in placenta of both groups. Smoking and non-smoking mothers and their newborns had similar MT levels in the serum and placenta. 

### 3.4. Association between Antioxidant Enzymes, MT and Elements

#### 3.4.1. Association of Cd and Pb with Other Elements

Cd and Pb were positively associated in all studied matrices (umbilical cord blood: Spearman’s r = 0.41, *p* < 0.001; maternal blood: r = 0.25, *p* = 0.03; placenta (only in smokers): r = 0.37, *p* = 0.03). Pb was positively associated with Mn (r = 0.26, *p* = 0.026) in cord blood, Cu in maternal serum (r = 0.30, *p* = 0.009) and with Fe in cord serum (r = 0.35, *p* = 0.002). In the placenta, Cd was positively associated with Cu (r = 0.34, *p* = 0.003), while Pb was positively associated with Zn (r = 0.29, *p* = 0.012).

#### 3.4.2. Associations between Antioxidant Enzymes and MT

GPx in maternal plasma showed a significant positive association with GPx in umbilical cord plasma (Spearman’s r = 0.55, *p* < 0.001), as well as SOD in maternal plasma with SOD in cord plasma (r = 0.50, *p* < 0.001). MT in maternal serum was significantly associated with placental MT (r = 0.41, *p* < 0.001). GPx was positively associated with SOD in both cord plasma (r = 0.25, *p* = 0.037) and placenta (r = 0.38, *p* = 0.001). Placental MT was positively associated with placental SOD (r = 0.55, *p* < 0.001) and GPx (r = 0.34, *p* = 0.003).

#### 3.4.3. Association of Cd and Pb with SOD, GPx and MT

Cd in maternal blood was negatively associated with GPx in maternal plasma (β = −0.07 [−0.13, −0.002], *p* = 0.044) after adjustment for age and education level. Similarly, placental Pb was negatively associated with SOD in placenta after adjustment for smoking status, age, and education (β = −0.06 [−0.11, −0.01], *p* = 0.019). However, only a minor amount of GPx and SOD variability was explained in these models (adjusted R^2^ values of 0.06 and 0.10, respectively).

#### 3.4.4. Identification of the Elements with the Strongest Impact on SOD, GPx and MT

According to the SDA analysis (Table S4), Zn and Fe in maternal serum, Mn in maternal blood and placental Pb were identified as the elements most strongly associated with SOD in maternal plasma. SOD in umbilical cord plasma was most strongly associated with Mn in maternal blood, Cd in cord blood, and placental Mn and Zn. For SOD both in maternal plasma and cord plasma, the results were inconclusive with respect to the directions of associations. SOD in placenta was most strongly associated with cord serum Cu and placental Zn, Se and Fe, where associations with placental Zn and Se were positive and associations with serum Cu and placental Fe negative. 

SDA analysis pointed to Fe in cord serum as negatively associated, and Se in maternal and cord serum and placenta as elements most strongly positively associated with GPx in maternal plasma. GPx in cord plasma was most strongly associated with cord serum Cu and placental Zn, Se and Fe (positive associations for all four elements), whereas GPx in placenta was most strongly associated with Mn in maternal blood, Fe in maternal serum, and placental Cd and Zn (inconclusive results with respect to the directions of associations). 

SDA analysis pointed to Pb in maternal blood, Cd in cord blood, Fe in cord serum and placental Mn as elements most strongly associated with MT in maternal serum. Directions of associations could not be determined from the SDA results. The level of MT in cord serum was most strongly associated with Zn and Se in maternal serum (positive associations) and placental Cu and Fe (negative associations). Placental MT was most strongly associated with Fe in maternal serum and placental Se, Fe and Mn. Only association with placental Fe was negative, while the other three associations were positive.

#### 3.4.5. Multiple Regression Models

Taking simultaneously into account four elements found significant from the preceding SDA analyses and predictors of age, education (and smoking) multiple regression analyses revealed significant associations with antioxidant enzymes or MT (Table 3). 

Higher values of placental SOD were significantly associated with higher placental Se values (*p* = 0.043) and lower placental Fe values (*p* < 0.001). The other two elements indicated by the SDA analysis, umbilical cord serum Cu and placental Zn, were not statistically significant predictors in the multiple regression model. The amount of variability of placental SOD values explained by the multiple regression predictors was relatively high, 67% (model *p* < 0.001, adjusted R^2^ = 0.67), mostly owing to a significant negative association of Fe and SOD in the placenta. On the other hand, SOD activities in maternal and cord plasma were not significantly associated with any of the considered predictors.

Predictors from the SDA model for GPx activity in cord plasma were cord serum Cu and placental Zn, Se and Fe. The multiple regression model revealed that higher values of GPx in cord plasma were significantly associated with higher values of placental Se (*p* = 0.014) and cord serum Cu (*p* < 0.001), while associations with placental Zn and Cd did not reach statistical significance. The included predictors explained for 24% of variation in cord plasma GPx values (model *p* < 0.001). Higher values of placental GPx activity were significantly associated with higher placental Zn (*p* = 0.019), while associations with placental Cd, Mn in maternal blood, and Fe in maternal serum were not statistically significant in the multiple regression model. Furthermore, only 3% of placental GPx variation was explained in the model and the model did not reach statistical significance. None of the predictors from the SDA model for GPx in maternal plasma (cord serum Cu and placental Zn, Cd and Se) was significantly associated with GPx in the multiple regression model.

Apart from Cd in umbilical cord blood and Pb in maternal blood, the model for maternal MT included Fe in cord serum and placental Mn. However, when these variables were entered into the multiple regression model with maternal age and education as confounders, none of these variables was significantly associated with MT. Higher values of MT in cord serum were associated with lower values of placental Cu (*p* = 0.031), while associations with Zn and Se in maternal serum and placental Fe were not statistically significant in the multiple regression model. Additionally, only a small amount of cord serum MT variation was explained in the model (2%; adjusted R^2^ = 0.02) and the model was not statistically significant. Multiple regression model for MT in placenta included Fe in maternal serum and placental Se, Fe and Mn. The higher MT values were in this model associated with higher values of Fe in maternal serum (*p* = 0.047) and lower placental Fe values (*p* = 0.002). Associations of MT with Se and Mn in placenta were not statistically significant. The included predictors explained for 23% of placental MT variability (model *p* = 0.001, adjusted R^2^ = 0.23).

## 4. Discussion

Cigarette smoking contributes to an increased production of ROS and increased exposure of mother and her foetus to various toxic chemicals, including toxic trace elements. Among the most abundant are Cd and Pb with proven toxic, genotoxic and carcinogenic potential. Both elements have a long half-life in the body, absorption rate that is about 10–20 times higher by inhalation than by gastrointestinal absorption, and numerous adverse health effects in virtually all organs in the body [7,34,35].

We studied the effect of active maternal tobacco smoking and related exposure to Cd and Pb on the antioxidant enzymes and MT in maternal and neonatal compartments considering the effect of the studied toxic elements on essential element levels (Cu, Fe, Mn, Se and Zn) and their role in antioxidant defence in the maternal-placental-foetal unit. Our results suggested that the effect of Cd or Pb on the antioxidant enzymes and MT in biological samples of maternal and foetal origin after controlling for other predictors in our study was not statistically significant, which is discussed in detail further on in the text.

In the present study, smoking postpartum women had higher Cd levels in blood and placenta compared to non-smokers, and smoking habit did not have an effect on Cd levels in newborns due to its expected limited placental Cd transport (Table S3). These results corroborate our previous findings in the studies on Cd exposure from cigarette smoke in healthy postpartum women [25,36,37]. In the present study, Cd levels in newborn cord blood were 4% and 9% of the levels in their smoking and non-smoking mothers, respectively. In other studied populations this percentage varied from 10% up to 79% (reviewed in [24,38,39,40,41,42]). As expected from the results of other studies [39,43,44], most of the absorbed Cd accumulated in the placenta, especially in smokers (Table S3). 

Our results opposed previous studies which found that smoking-related Cd in placenta increased placental MT expression [43,44], but was in line with our earlier study [23]. Discrepancies may be assigned primarily to 5-10 times higher placental Cd than in our participants, and different methods of placental MT quantification. ELISA was the method of choice in this study, Western blot in the study of Ronco et al. [43], Kippler et al. [39] and Phuapittayalert et al. [42]. A positive association of placental MT with food-derived Cd of non-smoking mothers with mean placental Cd of 130 µg/kg [39] and 35 µg/kg [42] placental Cd in these mothers from Bangladesh (*n* = 44) and Thailand (*n* = 14) were 5–17 times higher than median levels quantified in mothers in our study (7.50 µg/kg, Table S3) and high compared to literature data [45]. A low, but significant, difference in placental Cd between smoking and non-smoking mothers noticed here was obviously too low to result in visible changes in the MT level, either by induction of MT in placenta or redistribution of the Cd-MT complex already formed in maternal liver or kidney. Passage of Cd from mothers to newborns is restricted by binding to MT in the placenta, but this partial reduction and transport to newborns has been suggested to be orchestrated also by other molecular mechanisms including DMT1, ZIP14, ZnT2, CaT1, megalin, just to mention some [46,47,48,49]. Unbound Cd can cause toxic effects while interfering with Zn and Cu-dependent enzymes and membrane functions [49,50]. Alongside the fact that Cd failed to increase placental MT levels of mothers in our study, we also failed to notice any restrain in the Zn supply to newborns caused by binding to Cd-induced MT, corroborating previous reports [39,40].

In line with the reports of other authors [34,41], our results (Table S3) confirmed Pb passage through the placenta proposed to occur by passive diffusion [51]. Although in other populations Pb level in cord red blood cells or blood ranged 50–100% of that in mothers [24,38,41,52], according to our results, umbilical cord blood Pb was 69% of mothers blood. This result pointed to the placenta as an important, but rather poor barrier for Pb transfer from mother to newborn [53] enabling higher levels of maternal Pb than Cd to reach newborns [54]. Pb measured in the cord blood of our newborns (up to 7.68 µg/L) was below the currently determined level of concern for effects on neurodevelopment of 12 µg/L (95th percentile lower confidence limit of the benchmark dose of 1% extra risk, BMDL_01_; EFSA 2010). Therefore, we can assume that newborns in our study were not exposed to critical Pb levels at least in utero. 

Placental Pb showed more pronounced differences between smokers and non-smokers than Cd (Table S3), but in multivariate analyses (Table 3) Pb was not confirmed as a significant predictor of MT levels, in contrast to Cu and Fe. As Fe is a well-known inducer of oxidative stress [55,56,57], and MT was mentioned earlier to participate in free radical scavenging [56], we suggest that the associations found here reflect a free radical metabolism in the placenta. The association between MT and essential Cu can be addressed to the before mentioned mutual participation of MT and the antioxidative enzyme SOD (with Cu as cofactor) in maintaining oxidative balance. Co-expression of MT and Cu,Zn-SOD has been reported previously in yeast [58]. Contrary to our findings, Pb administration in rats was shown to result in MT production [59] following the mechanism of Pb-substituting Zn ions in MT and free Zn ions promoting the synthesis of new MT [60]. 

Metallothionein, except its main role in essential element storage and transport functions, also exerts a defensive role against free radicals [50,56,61]. Our results of a large surplus of MT in umbilical cord compared to maternal serum supports the need of newborn for Zn and Cu and free radical scavenger molecules as newborn lacks fully developed antioxidative mechanisms. However, it should be stressed that levels of MT in cord serum of our newborns were not associated with maternal or placental levels. Thus, we hypothesize that cord MT levels reflect both the MT transported from maternal serum and placenta, and the MT synthesized in newborns. MT expression was proven earlier in rat foetal liver [62]. MT and antioxidant enzymes have a common antioxidant role that was confirmed here through positive associations of MT levels with the activity of SOD enzymes in the placenta. Smoking as a sole factor (Table 2) or with other biological and environmental factors (e.g., maternal age, education) had no impact on MT levels (data not shown), in contrast to the reports by other investigators [24,43,63]. Those findings on induction of MT in maternal and cord blood and placenta of smoking mothers can be attributed to the enhancement of free radical production from metals and other free-radical inducing chemicals from cigarette smoke.

It has been suggested that smoking impairs the homeostasis of essential elements, although inconsistent results have been reported [13,19,23,25,36,64,65]. Smoking in our study contributed to lower Fe in maternal serum and placenta, Se in umbilical cord serum and placenta, and Zn in placenta when compared to non-smoking controls (Table S3). This is in line with the findings that smokers may have lower levels of antioxidants and essential elements than non-smokers as a consequence of decreased dietary intake or depletion of circulating antioxidant nutrient due to chronic tobacco smoke exposure [66,67]. Increased requirements for essential elements during embryogenesis, foetal growth and development often resulted in their decreased levels in maternal circulation, particularly if their intake through food was deficient. Most Fe in maternal circulation is used to expand erythrocyte mass, meet foetal Fe requirements and compensate for Fe due to blood loss at delivery. Therefore, decreased levels of Fe in maternal circulation during pregnancy are often present and also known as physiologic anaemia of pregnancy. Decreased Fe levels in the placenta of smoking compared to non-smoking mothers (Table S3) has also been found in our earlier study [25]. This may be connected with the increased levels of both Cd and Pb in placenta of smokers via competition for binding the same transporter proteins, e.g., divalent metal transporter 1 (DMT1). One of the possible indirect mechanisms of Cd-induced foetal toxicity is the enhanced binding of Zn and Cu to MT in the placenta leading to a reduced transfer of essential elements to the foetus [29,44,49]. We found no evidence to support this theory as Cd in the placenta showed no significant association with Zn or Cu in cord blood/serum, in contrast to findings by Kippler et al. [39] and Kantola et al. [64]. The inverse relation of Cd with Zn and Cu has been suggested to result from Cd-induced down-regulation of Zn (in placenta and maternal blood) and Cu transporters (in maternal blood) found in rodents [47,68]. However, we measured increased levels of placental Cu with increasing accumulation of Cd in placenta, which may involve the MT [50]. By sequestering Cu, MT hamper its involvement in redox reactions [50]. In line with the results reported by Tekin et al. [40], we did not find an association between Cd and Zn level in the placenta. 

When considering simultaneous exposure to selected elements in measured matrices, the results showed no significant influence of Cd or Pb on SOD and GPx variability (Table 3). The results for placental SOD suggested a strong influence of a decreased Fe and an increased placental Se. It was suggested that, in the conditions of Fe deficiency anaemia, increased SOD activity could be a compensatory factor for increased oxidative stress due to the shortening of the red cell life span and increased susceptibility to haemolysis [69]. The results for GPx in cord plasma suggested higher placental Se and cord serum Cu as significant predictors in multiple regression model. The obtained positive relationship between Se and GPx was expected since Se is present at the active site of GPx and changes in its concentration have an influence on the GPx activity. Accumulated placental Cd in smokers could form a biologically inert complex with placental Se, as was suggested in an experimental model [70], limiting Se transfer to the foetus and, hence, decreasing foetal (cord serum) GPx activity. As mentioned above, we found positive relationship between placental Cd and Cu. Elevated Cu can contribute to increased hydroxyl radicals that initiate lipid peroxidation [18]. GPx can effectively reduce reactive species by GSH. Therefore, elevated Cu may indirectly contribute to an activation of GPx, as was indicated in our study.

It has been suggested that series of free radical chain reactions in the organism of smokers contribute to the disruption of the dynamic balance between oxidation and antioxidation and depletion of antioxidant capacity resulting in decreased average erythrocyte activities of SOD, GPx and catalase (CAT) in smokers vs. non-smokers [71]. Active smoking during pregnancy depleted the antioxidant potential in maternal and cord plasma contributing to an impaired balance between the oxygen and nitrogen metabolism [12,13,14]. Higher erythrocyte SOD and GPx in smoking mothers in the third trimester of pregnancy (*n* = 28) with lower GPx in their newborns [72] and higher serum GPx in smoking mothers (*n* = 14) and their seven-days old infants with no difference in serum SOD at the postpartum seventh day [73,74] was observed when compared to non-smoking controls. In the present study, SOD activity was significantly lower in the maternal and cord plasma but significantly higher in the placenta of smokers when compared to non-smoking controls (Table 2). This SOD decrease in maternal and cord plasma and increase in placenta was parallel to the decreased Zn in maternal blood and increased Zn in placenta (Table S3) in smokers compared to non-smokers. The influence of Cd or Pb on SOD variability was not significant. The discrepancy between the results of other authors and our results could be due to the different recruitment times for the study populations, as well as to the different matrices used for enzyme activity analysis. We assume that increased oxidative stress due to active maternal smoking contributed to the inhibition of SOD activity in plasma. The resulting accumulation of superoxide radicals then induced expression of SOD observed as higher SOD activity in the placenta of the smoking mothers. Therefore, higher placental SOD activity may represent an adaptive response to a higher production of superoxide radicals. Higher SOD activity resulted in higher levels of hydrogen peroxide that must be efficiently neutralised by GPx and catalase. Thus, enhanced SOD together with enhanced GPx would contribute to the protection of the foeto-placental system against ROS toxicity [75,76]. However, we did not find a significant difference in GPx activity in any analysed matrices between the groups (Table 2). Overexpression of SOD can sensitize, rather than protect cells from oxidative stress and contribute to oxidative damage of DNA, proteins and lipids [77]. Impaired balance between antioxidant enzymes may have an important role for increased susceptibility of offspring to oxidative stress.

## 5. Conclusions

We found a significantly lower SOD in maternal and umbilical cord plasma, a higher SOD in the placenta of smoking compared to non-smoking participants, whereas GPx and MT did not differ between the study groups. Although active smoking during pregnancy contributed to higher placental Cd and Pb, their contribution to the variability of SOD, GPx or MT after control for maternal age, smoking, education and co-exposure to other elements was not significant. However, the impaired balance in antioxidant defence observed in the conditions of relatively low-to-moderate exposure levels to Cd and Pb could contribute to increased susceptibility of the offspring to oxidative stress and risk of disease development later in life. Further study on a larger number of subjects will help to better understand complex interactions between exposure to toxic elements and oxidative stress related to maternal cigarette smoking.

## Figures and Tables

**Table 1:** General characteristics of postpartum women and their newborns at delivery grouped by maternal smoking habit according to urinary cotinine levels ^1^.

	All (*n* = 74)	Non-Smokers (*n* = 37)	Smokers (*n* = 37)	*p* ^2^
**Maternal characteristics**				
Age (years)	31 ± 5	33 ± 5	30 ± 5	0.003
Education ^3^				<0.001
Primary school	4 (5.41%)	1 (2.70%)	3 (8.11%)	
Secondary school	33 (44.59%)	8 (21.62%)	25 (67.57%)	
University degree	35 (47.30%)	28 (75.68%)	7 (18.92%)	
BMI before pregnancy (kg/m^2^)	23.15 (14.69–36.59)	23.26 (18.37–36.33)	23.05 (14.69–36.59)	n.s.
BMI before delivery (kg/m^2^)	28.13 (19.83–41.52)	27.89 (22.57–41.52)	28.31 (19.83–38.64)	n.s.
Weight gain during pregnancy (kg)	14.29 ± 4.63	13.88 ± 4.18	14.70 ± 5.06	n.s.
Parity	2 (1–6)	2 (1–6)	2 (1–6)	n.s.
Urinary cotinine (ng/mL)	1730 (<LOQ–7723)	<LOQ	955 (101–7723)	<0.001
**Newborn characteristics**				
Male	52 (70)	26 (70)	26 (70)	n.s.
Birth weight (g)	3494 ± 496	3558 ± 486	3430 ± 503	n.s.
Birth body length (cm)	50.47 ± 2.02	50.59 ± 1.77	50.35 ± 2.25	n.s.
Birth weight/placental weight	8.74 (6.49–14.91)	9.19 (6.49–14.91)	8.23 (6.98–11.70)	0.017
**Placental characteristic**				
Trimmed weight (g)	401 ± 85.1	394 ± 90.2	408 ± 80.4	n.s.

^1^ Results are presented as mean ± SD, median (min-max) or number and percentage (%). Non-smokers had urine cotinine levels lower than limit of quantification (LOQ), smokers had urine cotinine levels ≥ 100 ng/mL. ^2^ Difference between smokers and non-smokers were tested by Student’s t-test, Mann Whitney *U*-test, or Fisher’s test and considered significant at *p* < 0.05. ^3^ Two smokers did not report their education level.

**Table 2:** Activity of antioxidant enzymes and MT concentrations in mother-newborn pairs ^1^.

	All (*n* = 74)	Non-Smokers (*n* = 37)	Smokers (*n* = 37)	*p* ^2^
**SOD (U/mL)**				
Maternal plasma	2.26 (2.00–2.67)	2.42 (2.18–2.66)	2.08 (1.87–2.60)	0.025
Cord plasma	3.74 (3.40–4.26)	4.13 (3.59–4.48)	3.64 (3.04–3.81)	0.004
Placenta	21.00 (8.09–39.52)	16.36 (6.14–32.65)	31.27 (12.44–43.38)	0.047
**GPx (nmol/min/mL)**				
Maternal plasma	69.76 (58.23–80.04)	70.22 (62.92–77.55)	63.60 (56.10–81.84)	n.s.
Cord plasma	41.33 (35.01–46.10)	41.50 (36.76–46.75)	39.90 (34.19–46.03)	n.s.
Placenta	20.83 (16.93–24.36)	20.36 (17.62–23.84)	21.00 (15.11–26.36)	n.s.
**MT (ng/mL)**				
Maternal serum	3.13 (2.46–4.01)	3.16 (2.43–3.55)	3.09 (2.56–4.21)	n.s.
Cord serum	35.14 (30.54–42.48)	34.03 (30.63–41.28)	36.63 (29.91–42.64)	n.s.
Placenta	178.5 (148.2–442.0)	174.6 (156.1–251.0)	197.8 (143.8–271.6)	n.s.

^1^ The results are presented as the median and 25–75% interquartile range. ^2^ Difference between smokers and non-smokers was tested by Mann Whitney *U*-test and considered significant at *p* < 0.05.

**Table 3:** Results of multiple regression analyses. Each row represents results of one multiple regression model with four elements identified from SDA analysis, smoking, age and education as predictors. Smoking was included as a predictor only in models without Cd as a predictor.

Dependent Variable	β [95% Confidence Interval]								*p*	Adj. R ^2^
	Intercept	Element 1	Element 2	Element 3	Element 4	Smoking	Age	Education		
**log(SOD_MP)**	0.680 [0.144, 1.216] *p* = 0.014	Fe_MS 0.133 [−0.022, 0.288] *p* = 0.092	Mn_MB −0.006 [−0.018, 0.006] *p* = 0.340	Pb_PL −0.017 [−0.036, 0.003] *p* = 0.087	Zn_MS 0.049 [−0.762, 0.861] *p* = 0.904	−0.052 [−0.210, 0.105] *p* = 0.508	0.007 [−0.007, 0.021] *p* = 0.296	−0.060 [−0.214, 0.095] *p* = 0.445	0.081	0.09
**log(SOD_CP)**	1.527 [0.865, 2.189] *p* < 0.001	Cd_CB −2.742 [−7.237, 1.753] *p* = 0.227	Mn_MB −0.010 [−0.021, 0.001] *p* = 0.085	Mn_PL 0.002 [−0.002, 0.006] *p* = 0.433	Zn_PL −0.010 [−0.066, 0.046] *p* = 0.716		0.001 [−0.010, 0.012] *p* = 0.851	−0.015 [−0.132, 0.102] *p* = 0.799	0.624	<0.01
**log(SOD_PL + 10)**	5.175 [3.489, 6.862] *p* < 0.001	Cu_CS −2.013 [−4.899, 0.874] *p* = 0.168	Fe_PL −0.017 [−0.020, -0.013] *p* < 0.001	Se_PL 0.007 [0.000, 0.013] *p* = 0.043	Zn_PL −0.024 [−0.131, 0.082] *p* = 0.650	0.082 [−0.173, 0.338] *p* = 0.521	−0.012 [−0.034, 0.009] *p* = 0.243	0.012 [−0.239, 0.263] *p* = 0.923	<0.001	0.66
**log(GPx_MP)**	3.203 [2.500, 3.905] *p* < 0.001	Fe_CS −0.001 [−0.036, 0.033] *p* = 0.944	Se_MS 0.002 [−0.004, 0.008] *p* = 0.526	Se_CS 0.004 [−0.007, 0.015] *p* = 0.479	Se_PL 0.004 [0.000, 0.008] *p* = 0.057	−0.030 [−0.188, 0.128] *p* = 0.705	0.005 [−0.008, 0.018] *p* = 0.473	−0.041 [−0.194, 0.112] *p* = 0.596	0.099	0.08
**log(GPx_CP)**	2.353 [1.645, 3.060] *p* < 0.001	Cd_PL 0.003 [−0.011, 0.018] *p* = 0.637	Cu_CS 2.726 [1.361, 4.090] *p* < 0.001	Se_PL 0.004 [0.001, 0.007] *p* = 0.014	Zn_PL 0.029 [−0.019, 0.078] *p* = 0.233		−0.002 [−0.012, 0.008] *p* = 0.708	−0.028 [−0.137, 0.080] *p* = 0.603	<0.001	0.24
**log(GPx_PL)**	2.374 [1.468, 3.280] *p* < 0.001	Cd_PL −0.006 [−0.028, 0.015] *p* = 0.550	Fe_MS −0.016 [−0.194, 0.163] *p* = 0.863	Mn_MB −0.006 [−0.021, 0.008] *p* = 0.371	Zn_PL 0.082 [0.014, 0.151] *p* = 0.019		−0.003 [−0.018, 0.012] *p* = 0.702	0.036 [−0.128, 0.199] *p* = 0.666	0.253	0.03
**log(MT_MS+5)**	1.946 [1.610, 2.282] *p* < 0.001	Cd_CB 0.625 [−2.811, 4.061] *p* = 0.718	Fe_CS 0.002 [−0.028, 0.033] *p* = 0.871	Mn_PL 0.003 [0.000, 0.005] *p* = 0.061	Pb_MB −0.006 [−0.015, 0.003] *p* = 0.211		−0.001 [−0.009, 0.007] *p* = 0.857	−0.007 [−0.092, 0.077] *p* = 0.868	0.382	<0.01
**log(MT_CP)**	3.845 [3.213, 4.477] *p* < 0.001	Cu_PL −0.403 [−0.767, −0.038] *p* = 0.031	Fe_PL −0.001 [−0.003, 0.001] *p* = 0.164	Se_MS 0.001 [−0.005, 0.007] *p* = 0.752	Zn_MS 0.186 [−0.628, 1.000] *p* = 0.650	0.039 [−0.100, 0.179] *p* = 0.575	0.003 [−0.010, 0.015] *p* = 0.654	0.047 [−0.095, 0.189] *p* = 0.512	0.338	0.02
**log(MT_PL)**	4.895 [3.859, 5.931] *p* < 0.001	Fe_MS 0.212 [0.003, 0.422] *p* = 0.047	Fe_PL −0.004 [−0.007, −0.002] *p* = 0.002	Mn_PL 0.004 [−0.001, 0.009] *p* = 0.149	Se_PL 0.003 [−0.002, 0.008] *p* = 0.259	0.117 [−0.092, 0.326] *p* = 0.268	-0.009 [−0.026, 0.009] *p* = 0.325	0.076 [−0.127, 0.280] *p* = 0.455	<0.001	0.23

Abbreviations: GPx—glutathione peroxidase, SOD—superoxide dismutase, MT—metallothionein, β—multiple regression coefficient, CB—cord blood, CP—cord plasma, CS—cord serum, MB—maternal blood, MP—maternal plasma, MS—maternal serum, PL—placenta. Smoking was coded as 0 for non-smokers and 1 for smokers; education was coded as 0 for primary and secondary school and 1 for university degree.

## Supplementary Materials

The following are available online at https://www.mdpi.com/2218-273X/10/6/892/s1, Table S1. Limit of detection (LOD) for metal quantification in human blood and results of the analyses of standard/certified reference materials used for quality control, Table S2. Limit of detection (LOD) for metal quantification in human placenta and results of the analyses of standard/certified reference materials used for quality control, Table S3. Concentrations of measured elements in maternal/umbilical cord blood and serum, and placenta grouped by maternal smoking habit, Table S4. Results of sparse discriminant analysis.
