# Supplementary material for: Cigarette Smoking during Pregnancy: Effects on Antioxidant Enzymes, Metallothionein and Trace Elements in Mother-Newborn Pairs
Source: Biomolecules. 2020 Jun 10;10(6):892. doi: 10.3390/biom10060892 (PMC7356311; doi:10.3390/biom10060892)
Supplement: Supplementary file 1 [file biomolecules-10-00892-s001.pdf]

Supplementary material

Journal: Biomolecules

Title: Cigarette smoking during pregnancy: effects on antioxidant enzymes, metallothionein and trace elements in mother-newborn pairs

Authors: Alica Pizent, Maja Lazarus, Jelena Kovačić, Blanka Tariba Lovaković, Irena Brčić Karačonji, Tanja Živković Semren, Ankica Sekovanić, Tatjana Orct, Karmen Branović-Čakanić, Nataša Brajenović, Andreja Jurič, Iva Miškulin, Lana Škrgatić, Sandra Stasenko, Tatjana Mioč, Jasna Jurasović, Martina Piasek

Corresponding author:

Maja Lazarus

Institute for Medical Research and Occupational Health

Ksaverska cesta 2, P.O. Box 291, HR-10001 Zagreb, Croatia

Tel: 00385 1 4682 538

Fax: 00385 1 4673 303

E-mail: mlazarus@imi.hr

Supplementary material contains: 4 Tables

Table S1. Limit of detection (LOD)[1] for metal quantification in human blood and results of the analyses of standard/certified reference materials used for quality control

Element		LOD whole blood	Seronorm™ Trace Elements Whole Blood L-1		Seronorm™ Trace Elements Whole Blood L-2		Seronorm™ Trace Elements Whole Blood L-3	
			Certified (mean (95% CI))	Measured (mean±SD)	Certified (mean (95% CI))	Measured (mean±SD)	Certified (mean (accept. range))	Measured (mean±SD)
Cd	µg/L	0.038	0.28 (0.17-0.40)	0.27±0.009	5.01 (4.00-6.20)	4.8±0.04	12.1 (10.8-13.4)	12.1±0.03
Mn	µg/L	0.226	18.4 (14.7-22.1)	19.2±0.40	31.4 (25.1-37.7)	32.0±0.47	47.3 (37.8-56.8)	47.5±0.55
Pb	µg/L	0.177	9.9 (7.9-11.9)	10.8±0.41	337 (269-405)	336±7.1	447 (401-493)	440±14.2
Element			**ClinChek® Whole blood Control L-1**		**ClinChek® Whole blood Control L-2**		**ClinChek® Whole blood Control L-3**	
			Certified (mean (range))	Measured (mean±SD)	Certified ((mean (range))	Measured (mean±SD)	Certified (mean (range))	Measured (mean±SD)
Cd	µg/L		1.23 (0.987-1.48)	1.26±0.054	2.88 (2.30-3.45)	2.73±0.066	6.32 (5.06-7.59)	5.93±0.465
Mn	µg/L		8.87 (7.09-10.6)	8.95±0.285	15.4 (12.3-18.5)	15.2±0.18	22.1 (17.7-26.5)	21.4±0.67
Pb	µg/L		54.5 (43.6-65.3)	55.5±0.79	219 (176-263)	225±5.8	425 (340-510)	421±1.6
Element		LOD serum	**Seronorm™ Trace Elements Serum L-1**		**Seronorm™ Trace Elements Serum L-2**			
			Certified (mean (95% CI))	Measured (mean±SD)	Certified (mean (95% CI))	Measured (mean±SD)		
Cu	mg/L	0.002	1.088 (0.999-1.176)	1.10±0.029	1.85 (1.7-2.0)	1.89±0.028		
Fe	mg/L	0.029	1.47 (1.17-1.77)	1.43±0.05	2.15 (1.72-2.58)	2.05±0.052		
Se	µg/L	0.057	87 (76-99)	87.6±0.69	138 (120-157)	131±4.8		
Zn	mg/L	0.010	1.097 (0.952-1.242)	1.11±0.02	1.617 (1.404-1.831)	1.61±0.009		
Element			**ClinChek® Serum Control L-1**		**ClinChek® Serum Control L-2**			
			Certified (mean (range))	Measured (mean±SD)	Certified (mean (range))	Measured (mean±SD)		
Cu	mg/L		1.06 (0.902-1.22)	0.98±0.004	1.39 (1.18-1.60)	1.36±0.046		
Fe	mg/L		1.09 (0.874-1.31)	1.09±0.034	1.73 (1.38-2.07)	1.79±0.042		
Se	µg/L		123 (98.1-147)	123±3.0	158 (126-189)	164±4.0		
Zn	mg/L		0.737 (0.626-0.848)	0.836±0.021	1.09 (0.926-1.25)	1.08±0.048		

[1]The limit of detection (LOD) was calculated as mean plus three times the standard deviation of a set of method blanks multiplied by dilution factor

Table S2. Limit of detection (LOD)[1] for metal quantification in human placenta and results of the analyses of standard/certified reference materials used for quality control

Element		LOD placenta	**IRMM BCR 185R Bovine liver**		**NIST SRM 1577b Bovine liver**		**IRMM BCR 186 Pig kidney**	
			Certified (mean (95% CI))	Measured (mean±SD)	Certified (mean (95% CI))	Measured (mean±SD)	Certified (mean (95% CI))	Measured (mean±SD)
Cd	µg/kg	0.023	544 (527-561)	530±21.2	500 (470-530)	511±28.6	2710 (2860-2560)	2678±27.3
Cu	mg/kg	0.002	277 (272-282)	255±18.1	160 (152-168)	165±5.5	31.9 (31.5-32.3)	31.0±0.81
Fe	mg/kg	0.047	-	163±23.2	184 (169-199)	192±15.7	299 (289-309)	277±36.2
Mn	µg/kg	0.889	11070 (10780-11360)	10616±732	10500 (8800-12200)	11070±88	8500 (8200-8800)	8233±586
Pb	µg/kg	0.026	172 (163-181)	169±11.2	129 (125-133)	139±2.7	306 (295-317)	299±11.5
Se	µg/kg	0.234	1680 (1540-1820)	1557±39.3	730 (670-790)	770±6.9	10300 (9800-10800)	9498±471
Zn	mg/kg	0.015	138.6 (136.5-140.7)	134±1.98	127 (111-143)	127±2.5	128 (125-131)	125±2.9

[1]The limit of detection (LOD) was calculated as mean plus three times the standard deviation of a set of method blanks multiplied by dilution factor

Table S3. Concentrations of measured elements in maternal/umbilical cord blood and serum, and placenta grouped by maternal smoking habit[1]

Element	All (N=74)	Non-smokers (N=37)	Smokers (N=37)	p^2
**Blood**				
**Cd_MB (μg/L)**	0.411 (0.286-0.723)	0.307 (0.242-0.351)	0.683 (0.544-1.205)	<0.001
**Cd_CB (μg/L)**	0.029 (0.020-0.037)	0.028 (0.019-0.034)	0.029 (0.024-0.038)	n.s.
**Mn_MB (μg/L)**	16.5 (12.8-19.9)	16.8 (12.4-21.6)	16.0 (13.2-18.6)	n.s.
**Mn_CB (μg/L)**	32.8 (27.2-44.0)	32.6 (27.7-43.0)	35.4 (26.3-44.0)	n.s.
**Pb_MB (μg/L)**	9.09 (7.32-11.0)	8.30 (6.56-10.9)	9.32 (8.08-11.4)	n.s.
**Pb_CB (μg/L)**	6.27 (4.99-7.68)	6.07 (4.50-7.37)	6.55 (5.22-8.10)	n.s.
**Serum**				
**Cu_MS (mg/L)**	0.628 (0.546-0.757)	0.598 (0.546-0.707)	0.649 (0.548-0.798)	n.s.
**Cu_CS (mg/L)**	0.173 (0.147-0.198)	0.175 (0.147-0.201)	0.171 (0.147-0.198)	n.s.
**Fe_MS (mg/L)**	0.896 (0.699-1.18)	1.06 (0.781-1.33)	0.760 (0.571-1.01)	0.002
**Fe_CS (mg/L)**	2.20 (1.82-2.65)	2.32 (1.82-2.81)	2.18 (1.82-2.54)	n.s.
**Se_MS (μg/L)**	51.5 (44.5-56.1)	52.1 (46.5-62.4)	48.9 (43.4-54.8)	n.s.
**Se_CS (μg/L)**	42.2 (38.2-46.0)	43.6 (39.9-49.5)	40.4 (37.0-45.1)	0.007
**Zn_MS (mg/L)**	0.487 (0.464-0.554)	0.487 (0.468-0.556)	0.484 (0.456-0.543)	n.s.
**Zn_CS (mg/L)**	0.814 (0.710-0.907)	0.816 (0.715-0.892)	0.813 (0.704-0.927)	n.s.
**Placenta**				
**Cd_PL (μg/kg wet wt)**	7.50 (5.63-9.94)	6.33 (5.45-8.59)	8.07 (6.03-11.9)	0.047
**Cu_PL (mg/kg wet wt)**	0.980 (0.899-1.08)	1.01 (0.905-1.09)	0.971 (0.897-1.07)	n.s.
**Fe_PL (mg/kg wet wt)**	104 (82.8-127)	109 (95.0-138)	94.4 (77.7-123)	0.021
**Mn_PL (μg/kg wet wt)**	82.3 (73.5-92.9)	81.5 (73.2-95.7)	84.0 (77.0-90.1)	n.s.
**Pb_PL (μg/kg wet wt)**	2.48 (1.86-3.67)	2.07 (1.62-2.99)	2.86 (2.24-5.07)	0.004
**Se_PL (μg/kg wet wt)**	160 (150-171)	162 (152-174)	158 (148-167)	n.s.
**Zn_PL (mg/kg wet wt)**	10.8 (10.3-11.7)	10.7 (10.1-11.5)	11.1 (10.6-12.2)	0.029

[1]Results are presented as the median and 25-75% interquartile range (in parenthesis). Abbreviations: MB–maternal blood, CB–cord blood, MS–maternal serum, CS–cord serum, PL–placenta

[2] The difference between smokers and non-smokers was tested with *t*-test in case of normal data distribution and Mann-Whitney test otherwise and considered significant at $p<0.05$.

Table S4. Results of sparse discriminant analysis. Prior to the analysis, all variables were transformed as described in section 2.3. For each dependent variable, a sparse linear combination was evaluated for each participant and results were summarized by the tertiles of the dependent variable. For variables where 1. tertile < 2. tertile < 3. tertile, positive coefficients were interpreted as positive associations, and negative coefficients as negative associations with a dependent variable. In cases where 1. tertile > 2. tertile > 3. tertile, positive coefficients were interpreted as negative associations, and positive coefficients as negative associations. For variables where the 2. tertile had the highest or the lowest median value, the results were interpreted as inconclusive with respect to directions of associations.

**Dependent variable**	**Sparse linear combination**	**Median [interquartile range]**		
		**1. tertile**	**2. tertile**	**3. tertile**
**SOD_MP**	0.12 Zn_MS - 0.31 Mn_MB + 0.96 Fe_MS -0.91 Pb_PL	-0.11 [-0.18, 0.03]	0.09 [-0.04, 0.12]	0.09 [-0.02, 0.19]
**SOD_CP**	0.02 Cd_CB - 0.02 Mn_MB + 0.18 Zn_PL + 0.09 Mn_PL	-0.01 [-0.02, 0.00]	0.02 [0.00, 0.03]	-0.01 [-0.03, 0.00]
**SOD_PL**	0.01 Cu_CS - 0.14 Zn_PL - 0.99 Se_PL + 5.41 Fe_PL	0.67 [0.45, 0.94]	0.07 [-0.26, 0.32]	-0.70 [-0.89, -0.43]
**GPx_MP**	-0.06 Se_MS - 0.07 Se_CS + 0.04 Fe_CS - 0.16 Se_PL	0.01 [-0.01, 0.03]	0.01 [-0.02, 0.03]	-0.01 [-0.03, 0.01]
**GPx_CP**	-2.63 Cu_CS - 0.44 Cd_PL - 0.08 Zn_PL - 2.36 Se_PL	0.42 [0.07, 0.57]	-0.01 [-0.28, 0.09]	-0.27 [-0.46, -0.07]
**GPx_PL**	0.32 Mn_MB - 0.43 Fe_MS + 0.23 Cd_PL - 0.86 Zn_PL	0.02 [-0.03, 0.11]	0.04 [-0.04, 0.14]	-0.07 [-0.12, 0.00]
**MT_MS**	-0.12 Cd_CB + 0.13 Pb_BM + 0.42 Fe_SP - 0.50 Mn_PL	0.01 [-0.04, 0.04]	0.02 [-0.01, 0.05]	-0.04 [-0.08, 0.02]
**MT_CP**	-0.11 Zn_MS - 0.31 Se_MS + 0.97 Cu_PL + 0.83 Fe_PL	0.06 [-0.05, 0.13]	0.05 [-0.05, 0.16]	-0.08 [-0.19, 0.04]
**MT_PL**	-0.01 Fe_MS - 0.06 Se_PL + 0.11 Fe_PL - 0.13 Mn_PL	0.01 [0.00, 0.03]	0.00 [-0.01, 0.02]	-0.02 [-0.03, 0.00]

Abbreviations: GPx–glutathione peroxidase, SOD–superoxide-dismutase, MT–metallothionein, β–multiple regression coefficient, CB–cord blood, CP–cord plasma, CS–cord serum, PL–placenta, MB–maternal blood, MP–maternal plasma, MS–maternal serum.
